# Metal Concentrations in Cosmetics Commonly Used in Nigeria

**DOI:** 10.1155/2013/959637

**Published:** 2013-12-05

**Authors:** Orish Ebere Orisakwe, Jonathan Oye Otaraku

**Affiliations:** ^1^Toxicology Unit, Faculty of Pharmacy, University of Port Harcourt, Rivers State, Nigeria; ^2^Department of Medical Laboratory Science, Faculty of Science, Rivers State University of Science and Technology, Port Harcourt, Rivers State, Nigeria

## Abstract

Trace amounts of potentially toxic metals can be either intentionally added to cosmetics or present as impurities in the raw materials. In the present study, the levels of lead, cadmium, nickel, chromium, and mercury have been assessed in 28 body creams and lotions, 10 powders, 3 soaps, 5 eye make-ups, and 4 lipsticks widely available on Nigerian markets. The increases over suggested or mandated levels of lead in these creams and lotions ranged from 6.1 to 45.9 and from 1.2 to 9.2 mg kg^−1^ when compared with Cosmetic Ingredients Review Expert Panel 2007 and German safe maximum permissible limit of lead in cosmetics, respectively. About 61% of the body cosmetics, the lotions, and the creams contained detectable levels of nickel ranging from 1.1 to 6.4–9.2 mg kg^−1^. Chromium and mercury were undetected in 100% of the cosmetic product. Taken together, lead and cadmium were high in creams and lotions. Most of the imported creams and creamy white coloured cosmetics contained higher levels of metal contaminants than the other colours. Regulatory Agencies in developing nations should take appropriate action for cosmetics that contain lead and cadmium beyond the reference limits.

## 1. Introduction

The quest for beauty has tended to promote the use of cosmetics by men and women. In spite of the profound interest in heavy metal hazards of cosmetics [1–6], very little attention has been given to metal contamination of cosmetic products in Nigeria and most sub-Saharan African countries [7]. According to Health Canada, 100% of all cosmetics product tested positive for nickel and over 90% tested positive for both lead and beryllium and on the average contained at least 4 of the 8 metals of concern (arsenic, cadmium, lead, mercury, beryllium, nickel, selenium, and thallium) [8]. Toxic metals content in cosmetic products is prohibited or at least restricted in regulations of many countries; however, the regulations are inconsistent and concentrations of metals permissible by particular regulations are different for various products and countries.

Humans are exposed simultaneously or consecutively to large numbers of chemicals of diverse structures from various sources and via multiple routes [9]. Cosmetics especially the skin lightening types are widely used in most African countries, especially by women. Since these products are used for long duration, on a large body surface area and under hot humid conditions, percutaneous absorption is enhanced [10].

The complications of these products can be serious. Some studies have documented an association between some ingredients of cosmetics and various health problems [9, 11, 12]. Females are at greater health risk in developing countries due to inadequate nutrition, unhealthy lifestyle, and environmental deterioration [13]. Physiological changes also can alter the bioaccumulation pattern of these metals in female body. Most of the metals act as endocrine disrupters interfering with female hormonal system [14].

The ever-glowing interest in cosmetics and the lack of their regulations in Nigeria necessitated this study. We have analyzed different types of cosmetics, using Atomic Absorption Spectroscopy, for the presence of lead, cadmium, nickel, chromium, and mercury and to quantify their concentrations. We have determined the fold-increases of these elemental impurities by comparing their levels with suggested/mandated levels of these metals. Since there seem to be no legislation regarding contaminants in cosmetic products in Nigeria and some other countries, we have employed German, Health Canada regulations, and available scientific studies in the interpretation of our data. The study will provide scientific data on the levels of these heavy metals to which an average Nigerian woman may be exposed from cosmetics.

## 2. Materials and Methods

Using a market basket protocol, fifty different cosmetics commonly used in Nigeria were bought from ten cosmetic shops in Port Harcourt, Rivers State, Nigeria, in March, 2011. The cosmetics included 28 body creams and lotions, 10 powders, 3 soaps, 5 eye make-ups, and 4 lipsticks.

They were aliquoted into sample bottles and labeled. About 0.2 g was weighed from each of the cosmetics samples, this was put into a digestion flask, and into each of the weighed samples was added 0.2–0.5 mL of the concentrated H_2_SO_4_ for wet ashing.

### 2.1. Method of Analysis

All measurements were carried out with a model analyst 800 Atomic Absorption Spectrometer (Perkin-Elmer, Shelton, USA) equipped with a Zeeman background correction system and an electrothermal atomizer with a transversely heated graphite tube. The wavelength for the determination of lead was 283 nm, cadmium 227 nm, chromium 356 nm, nickel 286 nm, and mercury 315 nm. Limit of detection (LOD) of AAS was 0.005 mg/L. The volume of each analysed sample was 20 *µ*L. Each sample was prepared twice and analysed in duplicate.

For quantitative analysis, five-point matrix-matched calibration curves were freshly prepared for each run in order to avoid interferences. We spiked five aliquots of 200 mg cosmetics or 50 mg petroleum gel with different amounts of metal.

Mercury was determined by the cold vapour technique after reduction with tin(II) chloride (SnCl_2_), in order to release the mercury in the sample solution. Precaution was taken at all times due to the toxic nature of mercury. A stock standard solution was prepared by dissolving 1.080 g of mercury(II) oxide, in a minimum volume of 1 : 1 HCL, and diluted to 1 litre with deionized water. This solution was then analyzed by the AAS using an air-acetylene, oxidizing (lean, blue) flame at a wavelength of 253.7 nm.

The concentration ranges based on cosmetics weight were 0.3–44.0 ppm for lead and nickel; 0.01–0.2 ppm for Cadmium; 0.03–0.4 ppm for mercury and chromium. Good linearity was obtained for all metals in the concentration ranges examined. The method detection limit (MDL) was defined as the concentration corresponding to three times the standard deviation (SD) of the average of seven blanks. The method gave MDLs (ppm) of 0.20 (lead), 0.006 (cadmium), 0.18 (nickel), 0.01 (chromium), and 0.02 (mercury).

The metals in the cosmetics were compared with Gondal et al. [15], and Basketter et al. [16] as they relate to cosmetics, Health Canada [8], Cosmetic Ingredients Review Expert Panel 2007 [17], and German safe maximum permissible limit [18] of cadmium to calculate the fold-increases over suggested levels of metals in the cosmetics.

## 3. Results

**Figure 1:**
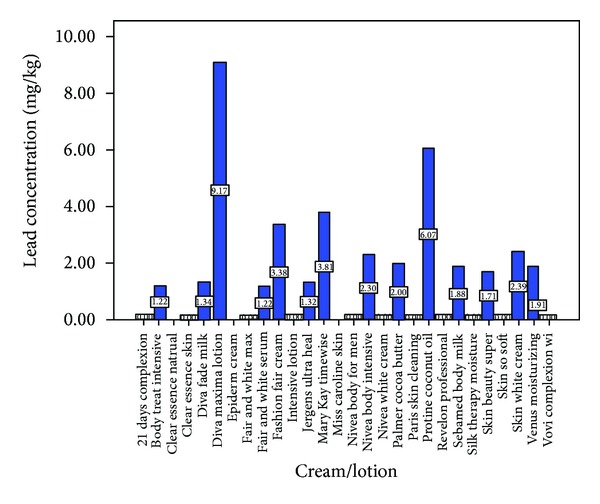
Lead concentration in creams and lotions. Suggested safe levels of Gondal et al. (0.5 ppm), Expert Panel 2007 (0.2 ppm), and Germany (1 ppm).

**Figure 2:**
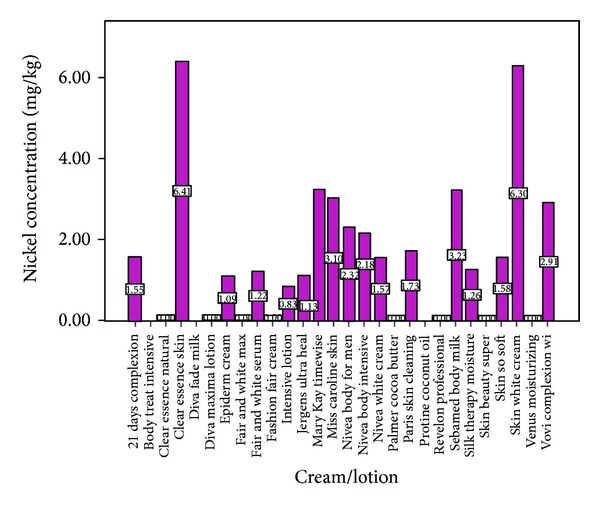
Nickel concentration in creams and lotions.

Figures 1 and 2 show the lead and nickel levels in creams and lotions used in Nigeria. Out of the twenty –eight different body creams and lotions fifty percent of this number contains detectable levels of lead. As chromium and mercury concentrations in the cosmetic products were below the detection limit there was no possibility to show them in the figures. The concentration of lead ranged from 1.2 to 9.2 mg/kg. The fold-increases over suggested/mandated levels of lead in these creams and lotions ranged from 2.4 to 18.3, 6.1 to 45.9, and 1.2 to 9.2 of Gondal et al., Cosmetic Ingredients Review Expert Panel 2007, and German safe maximum permissible limit of lead in cosmetics, respectively. Sixty-one percent of the body cosmetics, the lotions, and creams contained detectable levels of nickel. The concentration of nickel ranged from 1.1 to 6.4 mg/kg with fold-increases over suggested/mandated levels of 1.1–6.4. About fifty-four percent of these cosmetics contained detectable levels of cadmium. The cadmium concentration ranged from 0.2 to 2.3 mg/kg. The fold-increases over suggested/mandated levels of cadmium concentration in creams and lotions ranged from 0.3 to 4.6 of Gondal et al. safe maximum permissible limit of cadmium in cosmetics (figure not shown).

**Figure 3:**
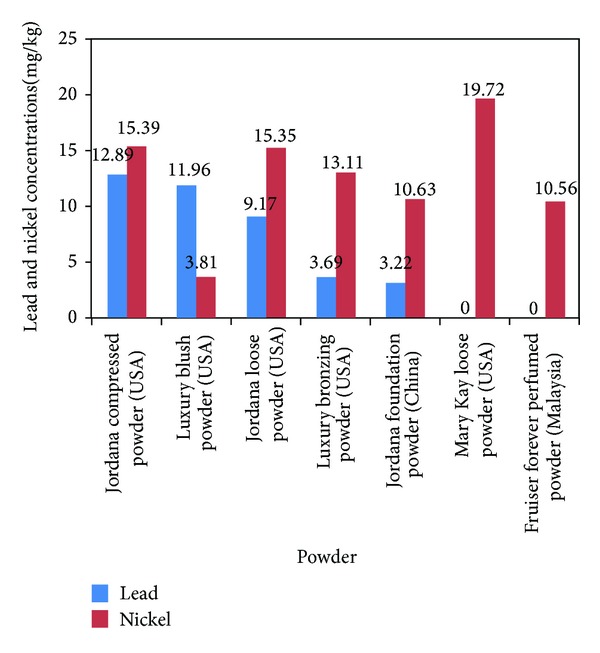
Lead and nickel concentrations in powders. Suggested safe levels of Gondal et al. (0.5 ppm), Expert Panel 2007 (0.2 ppm), Germany (1 ppm). Suggested safe levels of nickel: Gondal et al. (5 ppm), Basketter et al. low limit level (1 ppm), andBasketter et al. high limit level (5 ppm).

Seventy percent of the powders showed detectable levels of lead (range, from 1.3 to 12.9 mg/kg) (Figure 3). The fold-increases over suggested/mandated levels of lead in these powders ranged from 6.4 to 25.8, 16.1 to 64.5, and 3.2 to 12.9 times of Gondal et al., Cosmetic Ingredients Review Expert Panel 2007, and German safe maximum permissible limit of cadmium in cosmetics, respectively. One hundred percent of the powders had detectable levels of nickel. The nickel concentration and fold-increases over suggested/mandated levels range were 2.5–19.7 mg/kg and 2.1–3.9, 3.8–19.7 times of Gondal et al. and Basketter et al. safe maximum permissible limit of nickel in cosmetics, respectively. Detectable quantities (ranged from 0.18 to 2.21 mg/kg) of cadmium were seen in ninety percent of the powders with fold-increases over suggested/mandated levels of 2.2 to 4.4 times (figure not shown).

**Figure 4:**
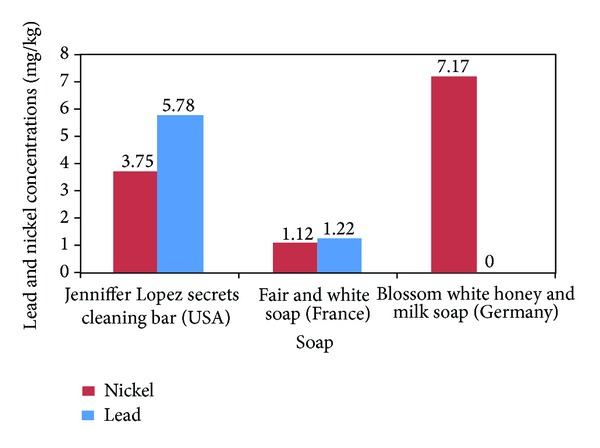
Lead and nickel concentrations (mg/kg) in soap. Suggested safe levels of Gondal et al. (0.5 ppm), Expert Panel 2007 (0.2 ppm), and Germany (1 ppm). Suggested safe levels of nickel: Gondal et al. (5 ppm), Basketter et al. low limit level (1 ppm), and Basketter et al. high limit level (5 ppm).

The levels of lead and nickel in different soap samples are shown in Figure 4. About 67% of the soaps contained detectable levels of lead that ranged from 1.2 to 5.8 mg/kg. The fold-increases over suggested/mandated levels of lead concentration in the soaps ranged from 1.2 to 11.6 times. Three soap samples had detectable levels of nickel with blossom white honey and milk soap showing high fold-increases over suggested/mandated levels. One hundred percent of the soaps contained detectable levels of cadmium. The concentration range was 0.14–0.83 mg/kg but the fold-increases over suggested/mandated levels of cadmium concentration in soaps were low (figure not shown).

**Figure 5:**
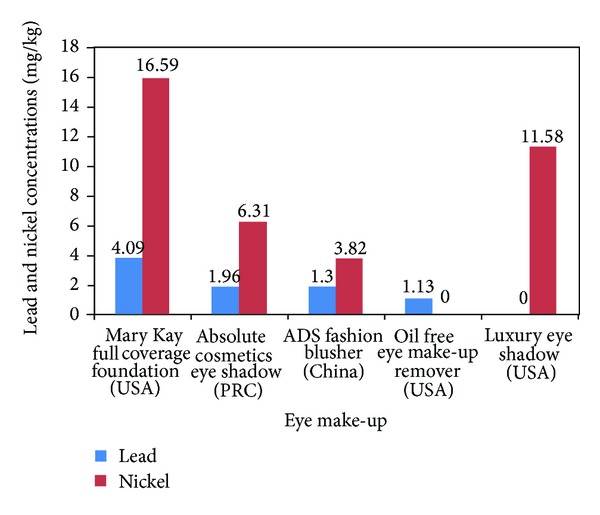
Lead and nickel concentrations (mg/kg) in eye make-ups. Suggested safe levels of Gondal et al. (0.5 ppm), Expert Panel 2007 (0.2 ppm), and Germany (1 ppm). Suggested safe levels of nickel: Gondal et al. (5 ppm), Basketter et al. low limit level (1 ppm), and Basketter et al. high limit level (5 ppm).

Figure 5 shows the lead and nickel concentrations in eye make-ups. Eighty percent of these eye cosmetics contained detectable levels of lead and nickel. The lead and nickel concentrations ranged from 1.1 to 4.1 mg/kg and 3.8 to 16.6 mg/kg, respectively. Lead showed the highest fold-increases over suggested/mandated levels of 20.5 whereas nickel had 1.3 to 16.6 times of fold-increases over suggested/mandated levels. Also eighty percent of the eye cosmetics contained detectable levels of cadmium with a concentration range of 0.2 to 3.5 mg/kg and fold-increases over suggested/mandated levels of 7 times (figure not shown).

**Figure 6:**
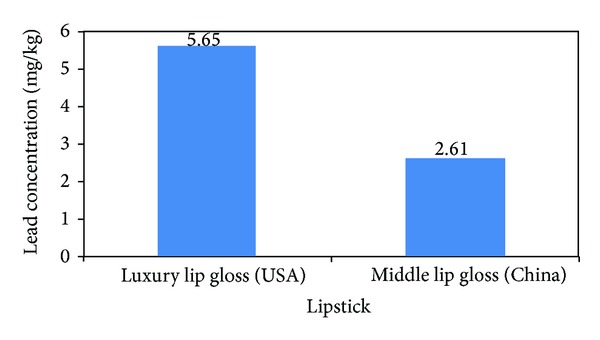
Lead concentration (mg/kg) in lipsticks. Suggested safe levels of Gondal et al. (0.5 ppm), Expert Panel 2007 (0.2 ppm), and Germany (1 ppm).

The lead concentrations in lipsticks are shown in Figure 6. Fifty percent of the lipsticks contained detectable levels of lead. Lead concentration in these cosmetics ranged from 2.6 to 5.7 mg/kg. The fold-increases over suggested/mandated levels of lead concentration in lipsticks are 2.6–28.3. Only Luxury lip gloss had detectable cadmium level that was however below the acceptable limit. Nickel was not detected in the lipsticks. Chromium and mercury were not detected in all the cosmetics studied.

In summary lead and cadmium were the highest in creams and lotions. Most of the imported creams and lotions had high fold-increases over suggested/mandated levels and creamy white coloured cosmetics contained higher levels of metal contaminants than the other colours. Brown and pink coloured imported powders and white soaps were more contaminated than other colours. Brown and blue coloured imported eye make-ups contained higher levels of metal contaminants than the other colours.

## 4. Discussion

Although cosmetics still retain their glittering appeal, their public health continues to mount. The US Food and Drug Administration (FDA) and European Union's Restriction on Hazardous Substances (ROHS) reported that some cosmetic materials used by humans contain hazardous substances [15]. Due to these reasons, there is a growing concern that some of the cosmetics may contain hazardous substances which are injurious to health [19, 20]. Although the use of metals as ingredients in cosmetics is prohibited in many advanced countries, metallic impurities cannot be avoided, even under good manufacturing practice, because they exist naturally in the environment [21].

This study has shown that some cosmetics used in Nigeria seem to be laced with lead of worrisome public health levels. A safe limit for lead is yet to be scientifically established, but many studies have indicated that it can be harmful at very low levels. Bellinger [22] showed that low levels of lead in women of childbearing age can have adverse effects on their reproductive health and/or offspring. The 2.6–5.7 mg/kg of lead seen in some imported lipsticks in this study may be of public health concern. Lead can be absorbed through the skin [23, 24] and although studies on the dermal absorption of lead in humans are still scarce, damaged skin, pH, and metallic chemical structure are likely factors that could enhance percutaneous penetration of metals [25, 26]. Tsankov et al. [27] determined lead contents in various cosmetic products and personal care products. The majority of their cosmetic products contained lead *≈*2.08 ppm. However, in only some decorative cosmetic, lead content was considerably high (*≈*41.1 ppm). Tsankov and colleagues [27] related their findings to an inadequate purification of the initial raw materials. Based on subacute dermal toxicity study on albino rats, Tsankov et al. [27] proposed that the maximum allowable concentration of lead should be *≈*10 ppm. If this permissible level is followed, only two of our tested cosmetics, namely, Dana compressed powder and luxury blush powder had lead contents >10 ppm. Another study by Sainio et al. [6] reported lead content (<20 ppm) in 49 eye shadow products.

Cosmetic products applied to mucous membranes are more hazardous, as in lipsticks and eye shadows [19, 20]. In addition to these risks, lipsticks may also have the higher risk of direct oral ingestion, aggravating the negative effects of their chemicals [19, 20, 28]. In view of these reasons and concerns, the analysis and evaluation of their effects on the human body are of importance. The main concern for safety assessment of use of these products is the precise knowledge about the concentrations and native hazards of the ingredients present in these products. Some of the species absorbed from the skin are systemically transported to different organs with attendant toxicities. The detected levels of lead and cadmium in lipsticks and other cosmetics in this study which are greater than the safe permissible limits of lead (15 ppb) and cadmium (5 ppb) in water and food may be of public health importance [29, 30]. It has been known for half a century that women may be more affected by cadmium than men. Bone disease associated with cadmium toxicity affected almost exclusively elderly, multiparous women [31]. Cadmium induced that decrease in the glomerular filtration rate was observed in women at lower exposure levels than those previously shown to affect male [32].

Lipstick use has been reported to be a risk factor and an environmental trigger for developing systemic lupus erythematosus (an autoimmune disease of unknown cause) [20, 28]. Although there is a possibility of genetic predisposition, discordance between identical twins and among dispersed people of the same ethnic background suggests that environmental factors are also contributors to disease expression [20]. Lead imparts the pigment in colored lipsticks. In the 33 popular brands of lipsticks tested by the Campaign for Safe Cosmetics, 61% were reported to contain lead with levels up to 0.65 parts per million, indicating a cause for concern [33]. Other studies that evaluated lead in eye shadows and lipsticks, namely, a U.S. Food and Drug Administration (FDA) study, detected lead in all tested lipsticks [2, 4, 34] and identified several cosmetic products containing Pb > 20 ppm, the FDA limit of Pb as an impurity in color additives for cosmetics. Studies carried out in other developing countries have also detected lead and cadmium in some lipstick samples [15, 35–37]. Like the work of Al-Saleh et al. [2] we also observed that coloured cosmetics like lipstick seem to have the high lead content. Although FDA does monitor lead levels in consumer products, levels above FDA limits can have detrimental effects, especially in sensitive populations like infants, children, pregnant women, and women of child bearing age. Although lead is absorbed very slowly into the body, its rate of excretion is even slower. Thus, with constant exposure, lead accumulates gradually in the body. It is absorbed by the red blood cells and is circulated through the body where it becomes concentrated in soft tissues, especially the liver and kidneys.

Since lead accumulates in the body over time, lead-containing cosmetics especially lipstick whether applied a number of times a day or on daily basis can contribute to significant lead exposure levels. Lilley et al. [38] suggested that lead absorbed through the skin may be eliminated via sweat and other extracellular fluids and hence not be as great a health hazard as ingested lead. There are few studies on lead dermal absorption. Gorter et al. [39] showed that no toxic risk was observed when commonly prescribed lead-containing ointment Plumbum metallicum 0.4% in humans was applied on skin.

In addition to the primary sources of lead exposure that Nigerians are exposed to, there seems to be recent studies and reports revealing the presence of lead in sundry consumables in Nigeria such as herbal remedies [40], beverages [41, 42], and drugs [43] that put the vulnerable population at risk of lead poisoning. To be exposed to lead from cosmetics may raise another concern. Njoku and Orisakwe [44] reported high blood lead levels amongst rural pregnant women in South Eastern Nigeria.

Pregnant women and young children are particularly susceptible to the dangers of lead exposure. The use of lead contaminated cosmetics especially lipstick or eye shadows by pregnant or/and lactating women could expose the fetus and infants to the risk of lead poisoning. Studies show there is no safe level of lead exposure [45]. Gilbert and Weiss [46] emphasized the importance of lowering the CDC blood lead action limit to 2 *µ*g/dL arguing that there is now sufficient and compelling scientific evidence showing that blood lead levels below 10 *µ*g/dL may impair neurobehavioral development in children. Lead has also been linked to infertility and miscarriage. Mendola et al. [47] examined published studies and research reports from 1999 to 2007 indexed in PubMed and found that exposure to lead is the strongest environmental contaminant that interferes with healthy reproductive function in adult females.

The nickel concentration and fold-increases over suggested/mandated levels range from 2.5 to 19.7 mg/kg and 2.1 to 3.9, 3.8 to 19.7 of Gondal et al.'s and Basketter et al. maximum permissible limit of nickel in cosmetics, respectively. Although it has been shown that the induction and elicitation of nickel contact allergy depended on the amount of nickel per skin unit area present in the epidermis [48] and a limit of 0.5 mg/cm^2^ per week of nickel release was suggested as a safe limit for nickel exposure [49], the concentration of nickel in creams and lotions from the present study which ranged from 1.09 to 6.41 mg/kg may be of adverse public health implications.

A number of metals and their compounds may cause adverse reactions upon contact with the skin [50]. Although some are toxic, causing irritant contact dermatitis, ulceration, or granuloma, the most common effect is contact allergy. Nickel is the most common cause of contact allergy of all skin sensitizers, and it is also an important cause of hand eczema. Sensitization to nickel is generally caused by direct and prolonged skin contact with items that release nickel ions. Depending on fashion, females of all ages—children, youths, and adults—are more exposed to nickel from such items than men. Therefore, nickel allergy is much more frequent in women than in men. Approximately 15–20% of the women and 2–5% of the men are allergic to nickel [51]. It is difficult to estimate whether a person not previously sensitized to nickel might acquire an allergy from the products studied. In individuals sensitized to these metals, concentrations as low as 1 ppm of nickel may cause an allergic reaction [52]. In this study nickel levels in powders, soaps, and eye make-ups were 3.8–19.7, 1.1–7.2, and 3.8–16.6 mg/kg, respectively.

Although it is known that polar organic compounds and some metals can appreciably penetrate damaged skin [24], there is limited knowledge about the behaviour of Ni metal powder. It is well known that the electrophilic nature of many metals, such as nickel, determines their protein reactivity, which can result in depot formation in the stratum corneum [53]. Such protein-metal binding can take place in all strata of the skin to the extent of building up a secondary barrier and inhibiting further diffusion [26]. The presence of nickel in cosmetics is forbidden by European Law 76/768/EEC [54], but their presence is permitted in very low quantities, defined as “impurities,” if it is “technically necessary.” However, there is no information about the amount of these metals that should be defined as an “impurity” and the methods to be used to quantify such traces. The scientific literature proposes a value for nickel lower than 5 ppm as “good manufacturing practice,” while the “target” amount to minimize the risk of sensitization in particularly sensitive subjects should be as low as 1 ppm [16, 52].

Interpreting how reported metal concentrations in cosmetics may be related to potential health risk can be challenging and it is usually not very easy to determine the contribution of cosmetics to the body burden of metals. It is worthy of note that cosmetics safety should be assessed not only by the presence of hazardous contents but also by comparing estimated exposures with health based standards [55]. Cosmetic with values above the safe limits should undergo a Health Hazard Evaluation to determine the level of risk posed by the product, which will then inform the appropriate enforcement action. Consumers cannot determine which products contain metals by reading the labels. It is the manufacturer's responsibility to ensure that the finished product contains as few heavy metal impurities as possible so that it does not exceed the limits. Regulatory Agencies in developing nations should request information on heavy metal test results for a cosmetic product if a risk is suspected. It is therefore in the manufacturer's best interest to have the information readily available. Regulatory Agencies in developing nations should take action as deemed appropriate for products that contain heavy metals beyond the reference limits. Manufacturers must ensure that their products and the ingredients used in the manufacture of their products are of high quality. This data indicates that the continuous use of these cosmetics may result in an increase in the trace metal levels in the ocular system and the human body beyond acceptable limits. The application of these cosmetics may be considered as a source of lead in evaluating patients with symptoms of lead intoxication in Nigeria and sub-Sahara Africa.
